# A Course of Lectures on the Diseases of Females

**Published:** 1843-11-11

**Authors:** 

**Affiliations:** Paris


### THE MEDICAL EXAMINER,

### And Retrospect of the Medical Sciences.

Vol. VI.] PHILADELPHIA, SATURDAY, NOVEMBER 11, 1843. [No. 22.

### A COURSE OF LECTURES

### ON THE DISEASES OF FEMALES,

*Delivered in the Summer of* 1843, *at the Hôpital L'Ourcine, (Veneriennes.)*

BY M. HUGUIER.

### LECTURE I.

### ON DERANGEMENTS OF THE PHYSICAL CONNECTIONS OF THE UTERUS.

In a physiological condition, and under ordinary circumstances, the uterus is in a manner suspended in the midst of important viscera, without its presence being indicated by any inconvenience to the neighbouring organs. When by any cause, however, the uterus changes its situation, by direction, or in volume, this *consensus*, this harmony, is instantly destroyed, and derangements, more or less extended, *malaises*, and even accidents, may result from this alteration of position. In such cases are observed particular symptoms, which are not caused by the functions of the uterus themselves, but by those of the neighbouring organs. If there be anteversion, for instance, the patient experiences a sensation of heaviness, produced by the pressure of the uterus upon the posterior part of the bladder; from this, vesical irritation results, with constant desire to evacuate the urine, although the bladder contains but a small quantity; there is, besides, more or less severe pain in the region of the bladder, with a peculiar uneasy sensation, which, however, is not the chief seat of these disorders. In cases of pregnancy, or of voluminous polypus of the uterus, or other affections in which this organ is greatly developed, the vesical derangements are frequently carried very far.

In cases of retroversion, the uterus, on the contrary, presses on the rectum; the patient complains of a feeling of uneasiness, of heaviness in that region, with a perpetual desire to go to stool. The efforts made to obey this call augment, instead of diminishing, the pain. Under some circumstances, in cases of increase in the size of the uterus, either by simple hypertrophy of the organ itself, or by the accumulation of fluids in its cavity, or the presence of a polypus, or in pregnancy, this pressure becomes the cause of more or less obstinate constipation.

It is, above all, in cases of augmentation of volume of the uterus by one of the causes which I have just summarily indicated, that change in the situation and connections of the uterus, determine accidents which become often formidable. You may have, for example, compression of the pelvic nerves, of the great sacro-sciatic plexus, and as a consequence pain in the abdominal cavity, in the whole pelvis, in the lumbar region, and in the groins; genuine neuralgia may supervene, either in the crural nerve, or in the sciatic nerve. These occurrences are by no means rare. You will find several mentioned in the "Clinical Surgery" just published by M. Lisfranc. We have now, in the Salle St. Alexis, a patient that I shall bring before you, who is suffering from cancer of the uterus. What is remarkable in her case, and which is worthy of attracting your notice, is the fact, that she has but little pain in the parts which are the seat of the disease, but intense pain in the left limb. The truth of this assertion you can substantiate when the patient comes before you.

Very often compression of the lymphatic vessels and glands occurs, and you will observe in many women, as a consequence of disease of the genital organs, œdema of the lower extremities, of the greater and lesser labia, and of the entire vulva, parts which are not the seat of the principal and primary affection. These special symptoms may go so far as to produce paralysis of the lower extremities, for any cause which produces enlargement of the uterus, may produce pressure on the nerves, and paralysis will follow. M. Lisfranc, in the work I mentioned, cites two examples; in these cases the paralysis lasted for more than eight years, and had resisted the most energetic anti-neuralgic treatment; it yielded to a treatment directed against engorgement of the uterus.

These are by no means the only lesions which can result from derangement in the physical connections of the uterus; it is not uncommon to see the uterus in a hernial tumour; it is chiefly in cases of crural hernia that this occurs. The ovaries are sometimes in the same predicament. The uterus thus displaced drags the bladder with it. The same thing may happen when there is abdominal eventration, in women who have borne a great many children.

It is important to know these facts, in order that the surgeon may not mistake one affection for another entirely different, and which is produced by lesions having their seat in other organs.

It sometimes happens that the uterus, after having risen to some distance in the cavity of the abdomen, contracts adhesions, as I have often seen, (and I am going to exhibit to you a very curious case,) with the small intestines, the arch of the colon, its sigmoid flexure, the superior extremity of the rectum, or with the omentum majus, as shown by a very remarkable specimen which I possess. If it happens that the uterus, thus occupying a region which is not natural to it, regains its natural size, and redescends to its primitive position in the pelvis, or even lower down still, dragging feelings will result, pains in the bowels, caused by the displacement, and derangements in the functions of the intestines. Laceration of these adhesions even may occur, or of the parts between which they are formed. These adhesions very commonly occur with the superior part of the posterior face of the bladder, and disturbances in the functions of this organ supervene. From this it follows that if the physician does not employ touching, he is unable directly to appreciate the lesion which exists, as well in relation to its seat as to its nature, and he may be led to treat a patient for a disease which does not exist. When, on the contrary, he has ascertained the species of lesion which is the cause of the functional disorders he observes, he applies a pessary, for example, to replace the uterus, and fix it in a position sufficiently elevated to avoid those accidents which it might produce without these precautions.

It sometimes happens that the uterus, though perfectly healthy, falls towards the vulvar aperture, and presses upon the inferior orifice of the vagina. In such cases there is felt a weight, together with vague pains; sometimes a feeling of distension, as if a foreign body were ready to escape through the orifice; there is often, besides, towards the groin, an unusual pain; at other times, the functions of the bladder and rectum are deranged. In anteversion of the uterus, when it is, as I said before, drawn over the pubis, the bladder often follows the genital organs in their change of direction. From this there results, also, change in the relations, the form, and situation of the urethra, its orifice being found above, or behind, its natural position, towards the posterior part of the sub-pubic ligament, and thence derangement in the functions of the excretory apparatus for the urine, although not itself, properly speaking, the seat of any direct lesion.

All these varieties are the more important to know, for if not understood, you might be led to believe in the existence of disease of the bladder, when there is only simple subsidence of the body of the uterus; and these functional derangements are the more fallacious, and liable to lead into error, as nothing, very often, announces the derangement, or the lesion of the uterus, its functions all being performed very regularly. Thus menstruation, conception, gestation, continue to be accomplished with the greatest regularity, as well as the other acts appertaining to the organs which compose the sexual system. What is there more natural or more rational than to consider as the seat of disease the organs which are the seat of the pathological phenomena, and to regard as healthy those parts which are the seat of no pain, and whose functions are performed, or appear to be performed, regularly? I could cite to you innumerable instances of grave errors which have been, and are daily being, committed from no other causes than those which I have just enumerated. Not only may the affections of the uterine system embarrass the physician little skilled in their study, and simulate disease of the stomach, of the small intestines, of the bladder, or of the rectum, but the pathological condition of these two last reservoirs can, in its turn, produce morbid phenomena, whose real seat is some portion of the genital apparatus. I recollect having twice seen venous metorrhagias extremely abundant, which were caused simply by the pressure which a rectum, dilated by an accumulation of hardened fœcal matter, exercised upon the uterine system,—hemorrhagias which ceased, as it were, by enchantment, when, by the aid of purgatives, evacuation of the intestines were produced. At other times, the matters accumulate in the superior extremity of the rectum, in the sigmoid flexure of the colon, and form a tumour, which, applied to the posterior surface of the uterus, compresses this organ and depresses it. If you examine a woman under these circumstances, the finger introduced into the vagina discovers the prolapsus of the uterus, but shows that the os tincæ is sound. Carried towards the posterior surface of the uterus, it encounters a hard body, often irregularly rounded, which seems to be continuous with it, and to form part of its substance. The practitioner will be liable, if not very careful and attentive, to mistake this body for a tumour, or engorgement of the posterior parietes of the uterus. This error is still more easy, as it sometimes happens, under analogous circumstances, that adhesions are formed between the superior border, or the posterior face, of the uterus, and the anterior wall of the rectum, and that, by this means, the rectal tumour is intimately connected with the uterus. You will avoid with still greater difficulty this error, if the tumour is of ancient date, and formed of very hardened excrementitial matter. The more liquid portion of the fæces will have found a canal in the midst of the stercoral tumour, through which they arrive at the anus. In this instance, defecation being regular, the attention of the physician is not called to the point which should attract his notice. When touching is practised without the most scrupulous attention, you may believe in an engorgement in the posterior part of the uterus, which is, notwithstanding, sound. I have seen this error frequently committed, and that, too, by practitioners of great merit. M. Lisfranc acknowledges himself having once committed it.

On the other hand, when the bladder is the seat of retention of urine, it can depress, displace the uterus, change its direction, and produce accidents which are, in reality, only accessory and consecutive phenomena, and which one should be careful not to consider as essential.

It sometimes happens that a simple relaxation of the parts is mistaken for prolapsus of the vagina, or hernias of the vagina, or of the uterus. Thus, in women who have borne a great number of children, in whom the vagina is very large, and the vulvar opening very much dilated, the bladder and the rectum, as much from weakness of the recto-vaginal and vesico-vaginal partitions, as from the want of support that they experience from the parts corresponding to the vulvo-uterine canal—from these causes, the bladder and rectum, I say, form a prominence, form tumours, more or less voluminous, which, during the repletion of these reservoirs, and the efforts of expulsion which the woman makes, augment in volume considerably, drag with them the corresponding part of the vagina, and fall through the vulvar ring, so as to simulate a prolapsus of the vaginal parietes, or even of the uterus; for it often happens that this organ itself is forced down and drawn towards the orifice of the vulva. You must still be very guarded how you consider these utero-vaginal affections as primitive. They are but consecutive to dilatations of the anterior parietes of the rectum and of the bas-fond of the bladder. Hence the explanation of the insufficiency of the operations which, up to this time, have been performed upon the vagina for the cure of these infirmities.

The utero-vulva canal is so disposed in its relations with the uterus, that it cannot and ought not, in a healthy state, to give passage to anything except the fluids secreted by the vagina or uterus,—the mucous, purulent, or sanguine discharges, coming either from the canal itself, or from the chief organ of generation. So soon as you perceive flow from its orifice other matters than these, such as stercoral or urinous matters, calculi, whether vesical or biliary, you may be assured that the physiological connections and relations which exist between the genital apparatus, are disturbed, and that an anormal communication is established between parts where naturally none should exist. And yet here you have the symptoms of a functional lesion of these organs.

*Derangements in the natural relations of different portions of the genital system.*

In some women you meet with an anormal development of the clitoris—a development which produces, as a consequence, an enlargement equally anormal and considerable, proportioned to that of the clitoris, of the nymphæ, which should form its preputial covering. In such cases the nymphæ extend beyond the greater labia, between which they hang; a disposition which you should not take for a morbid development or accidental hypertrophy.

In others, again, the vagina is very large; its parietes are dilated and weakened beyond conception; in such instances the canal loses in length what it gains in width. From this there results a subsidence of the uterus, as a necessary consequence, and which ought not to be considered as an essential phenomenon. It is in similar cases, when there is some hope of curing this infirmity, that the treatment should not be directed to the uterus, but to the vagina, which alone, by its vicious disposition, has determined the production of the accident.

In the diseases of the ovary, as soon as the tumour acquires a certain volume, it does not fail to impress upon the uterus changes in its situation, its direction, and forces it, or drags it, into one of the iliac fossæ. Its functions are equally altered and modified, although the organ itself is perfectly sound; hence you frequently see ovarian disease mistaken for an uterine affection. Under these circumstances, if you examine the patient by the touch, you will find the uterus higher up than natural. Its direction is changed, but these modifications in its natural situation and direction are only consecutive. It is a secondary and concomitant affection, induced by the intimate connexions and anatomical relations existing between the uterus and ovary.

Let us now examine the functional relations between the genital organs and the neighbouring parts, and those between the different parts of this apparatus itself. There is, in the whole sexual apparatus, a mutual dependence. The various acts, whose ensemble constitutes the great and important function of reproduction, are so united, so intimately linked, one with the other, either directly or indirectly, that when one of them fails, the principal end of nature, the reproduction of the species, is attacked. You may conclude, from non-fecundation, either that one of the organs charged with the generative function is wanting, or that they are under the influence of some morbid influence. When a young girl reaches the age of nubility, and you do not see appear in her the physical characters of her sex, if the breasts do not increase in size, if the pelvis is not developed, if the sacro-vertebral curve is not sensibly manifested; when you do not see menstruation established, and the desire and want of sexual relations occurring, you may conclude, either that the organs of generation in this case have not reached their maturity, or that some fundamental portion of the uterine system is wanting, as the uterus, ovaries, &c. If, in a woman presenting all the external characters of her sex, and in whom the menstrual evacuation occurs with regularity, sexual desire does not exist, or if connection cannot be effected, you may at once consider as established one of two things—viz., either that some part of the genital apparatus—the vulva or vagina—is the seat of some vice of conformation, or of a spasmodic contraction which prevents the entrance of the viril member, or that there is excess of sensibility. You sometimes meet with young girls whose vaginas are endued with excessive sensibility, to such a point that the finger, or a mesh well anointed, cannot penetrate, and are forced outwards by contraction of the vaginal parietes. To overcome this sensibility, you should employ soothing means, and a progressive dilatation. Consulted once in a case of this kind, I performed upon the vagina a little operation that is ordinarily done in fissure of the anus. The act of copulation accomplished, if it be not followed by fecundation, you must seek further for the cause of sterility. You must then suspect an anormal disposition of the uterus, or ovaries, or some profound alteration in these parts.

*Paris, September*, 1843.

